# Internal Medicine Acting Internship Trends in Rotation Structure and Student Responsibilities: Results from a 2023 National Survey

**DOI:** 10.1007/s11606-024-08897-2

**Published:** 2024-07-29

**Authors:** Adam M. Garber, T. Robert Vu, Andrew Orr, William Adams, Irsk Anderson, Matthew Fitz, Allison Ferris

**Affiliations:** 1 Virginia Commonwealth University School of Medicine, Richmond, VA USA; 2 Indiana University School of Medicine, Indianapolis, IN USA; 3 San Francisco VA Medical Center, University of California San Francisco School of Medicine, San Francisco, CA USA; 4 Loyola University Stritch School of Medicine, Maywood, IL USA; 5 University of Chicago Pritzker School of Medicine, Chicago, IL USA; 6 Florida Atlantic University Schmidt College of Medicine, Boca Raton, FL USA

## Abstract

**Background:**

The acting internship (AI) in internal medicine plays a key role in the transition from medical school to residency. While there have been recent changes in medical education including a pass/fail USMLE Step 1 and increasing use of competency-based assessment, there has not been a large survey of the state of the AI in many years.

**Objective:**

To assess the current landscape of the internal medicine AI and identify areas in need of standardization.

**Design:**

This was a voluntary online survey of medical schools in the United States (U.S.).

**Participants:**

Course directors of the AI rotation at U.S. medical schools.

**Main Measures:**

Number of AI rotations required for graduation, length of AI rotation, types of services allowed for AI, clinical responsibilities of students, curricular components.

**Key Results:**

Response rate was 50.7% (71/140 LCME accredited schools). All responding institutions require at least one AI for graduation, with nearly all schools integrating students into resident teaching teams, and almost half also allowing AI students to work on hospitalist services. Students carry 3–4 patients per day on average with a maximum of 5–6 in most institutions. Students are responsible for most aspects of patient care including notes, orders, interprofessional communication, and transitions of care. Night call or night float responsibilities are infrequently required. The structured curriculum published by AAIM is used by only 41% of schools.

**Conclusions:**

The internal medicine AI continues to be a staple in the medical school experience, but there is variation in the structure, curriculum, and expectations on the rotation. Opportunities exist to improve standardization of the AI experience and expectations to better prepare medical students for the transition from medical school to residency.

**Supplementary Information:**

The online version contains supplementary material available at 10.1007/s11606-024-08897-2.

## INTRODUCTION

Despite calls for transformation, the current transition from medical school to residency remains, as described in a recent root cause analysis, “dysfunctional.”^1–3^ Stakeholders cite a lack of alignment between undergraduate medical education (UME) and graduate medical education (GME) as one contributing factor to this dysfunction.^4^ Residency program directors (PDs) already struggle to quickly glean evidence of an applicant’s ability to succeed in residency. They now face additional challenges in the era of pass/fail licensing exams,^5,6^ as well as the trend towards pass/fail grading at some schools. In the wake of these changes, PDs reviewing residency applications believe they will place greater emphasis on performance and experiences from clinical rotations, including the fourth year acting internship (AI; also known as “subinternship”).^7,8^

The AI in internal medicine, with its focus on practical intern year skills such as time management, task prioritization, and transitions of care, specifically offers crucial data for the UME to GME transition.^9,10^ However, variability in rotation design across institutions and incomplete or outdated data from prior national surveys have historically limited transparency and hindered the course’s usefulness on a national level.^11^ Further, existing data in this realm, including the most recent survey about the AI in 2017 by the Alliance for Academic Internal Medicine (AAIM), predate the AAMC’s Core Entrustable Professional Activities (EPAs) and likely do not reflect the recent advances and increased complexity of the internal medicine AI.^12–16^ Core EPAs have emerged as important to IM PDs^17^ and some medical schools have begun to adopt these into their curricula,^18,19^ yet there is a lack of uniformity about the AI rotation curricula and structure nationally. Given this discordance, we developed a survey to identify areas in need of standardization, and in turn to further fortify the role of the medicine AI as a pivotal bridge in the UME to GME transition.

## METHODS

The study team conducted a literature search and used prior publications and surveys from AAIM to develop survey questions which would capture data about the current state of the IM AI. The authors tested the questions with colleagues having a background in survey development and subsequently refined the questions prior to building the survey in REDCap hosted at Florida Atlantic University (see Supplemental Material).^20,21^ In January 2023, the authors used the Clerkship Directors in Internal Medicine (CDIM) listserv to post a request to complete the survey (with a link to the REDCap survey). Repeat reminders were posted at the 3-, 4-, and 6-week marks, after which time authors sent personal emails to colleagues from non-responder institutions. After 8 weeks, the survey closed. For schools that submitted multiple surveys or had incomplete submissions, study personnel personally contacted colleagues at those schools to obtain clarified and/or more definitive answers to the survey.

Descriptive statistics were used to summarize demographic data. Exact one-sample two-sided chi-square tests were used to assess whether responses for the current study differed from historical rates. All analyses were completed using SAS version 9.4 (Cary, NC).

The Florida Atlantic University Institutional Review Board deemed this study exempt from ethical review.

## RESULTS

Seventy-one participants representing different institutions completed the survey for a response rate of 50.7% (71/140 LCME schools). Compared to past CDIM national survey results, our survey respondents mirrored the same breakdown in institution class sizes and similar geographic regions. Regarding school class size, most respondents fell into the 121–200 students per class. See Table 1 for demographics of respondent characteristics and AI structure.

**Table 1:** Survey Respondent Demographics

Survey question prompt	Answer choices	% response (*n*)
**Class Size**	1–50	2.8% (2/71)
	51–80	8.5% (6/71)
	81–120	19.7% (14/71)
	121–200	53.9% (39/71)
	> 200	14.1% (10/71)
Region*	Northeast	26.9% (18)
	South	29.9% (20)
	Central	29.9% (20)
	West	13.4% (9)
**# of AI rotations required for graduation**	1	64.7% (46/71)
	2	28.2% (20/71)
	3 +	7.0% (5/71)
**Does your school require an AI in internal medicine for all students (regardless of planned specialty)?**	Yes	10% (10/70)
	No	90% (63/70)
**If you require an AI, can an away rotation fulfill the AI requirement?**	Yes	22.9% (16/70)
	No	67.1% (47/70)
	Unsure	10% (7/70)
**Length of AI**	4 weeks	98.6% (70/71)
	8 weeks	1.4% (1/71)
**Setting of AI**	Entirely inpatient	94.4% (67/71)
	Equal mix inpatient/outpatient	4.2% (3/71)
	Primarily inpatient w/some outpatient	1.4% (1/71)
**Structure of the IM AI rotation**	Integrated onto teaching service w/residents/interns	94.4% (67/71)
	With hospitalist only	42.3% (30/71)
	With outpatient faculty only	1.4% (1/71)
	Outpatient teaching clinic with residents/interns	1.4% (1/71)

*Based on U.S. Census regions per prior CDIM surveys; 3 respondents’ regions unknown

### Structure and Graduation Requirements

The majority of IM AI rotations are 4 weeks in duration (98.6%, *n* = 70) and are predominantly inpatient-only (94.4%, *n* = 67). Overall, 94.4% (67/71) of institutions integrate students into traditional teaching services with residents/interns, while 42.3% (30/71) also offer an AI in which students are integrated into hospitalist-only services.

**Figure 1:**
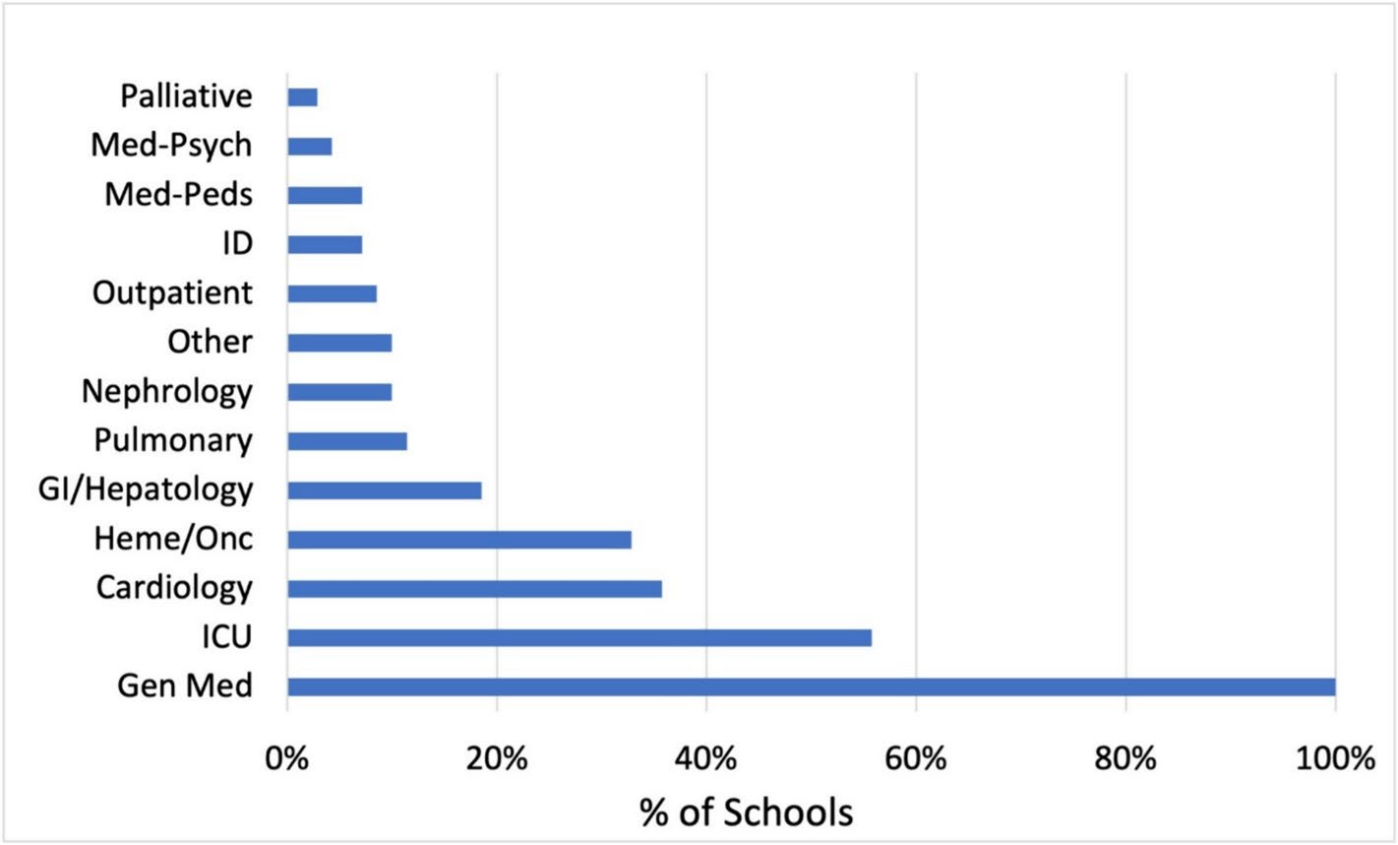
AI rotation clinical settings and services.

The number of settings and specialties on which an AI rotation is offered is highlighted in Fig. 1. While nearly all schools offer a general medicine AI experience (98.6%, *n* = 70), there are multiple options for students to complete their AI rotation, including ICU (54.9%, *n* = 39), Cardiology (35.3%, *n* = 25), and Hematology/Oncology (32.4%, *n* = 23).

All respondents (*n* = 71) require at least one AI rotation as a graduation requirement with 22.9% (*n* = 16) of respondents noting their institution allows an away AI rotation to fulfill that AI requirement. Sixty-five percent (*n* = 46) of institutions require one AI while 28.2% (*n* = 20) require two AI rotations for graduation, and 7.1% (*n* = 5) require three or more AIs. Despite only 10% (*n* = 7) of respondents reporting their institution specifically requires an AI in IM for graduation, nearly two-thirds (64%, *n* = 45) of respondent schools report that greater than 40% of their students complete the IM AI regardless of the requirement.

### Curriculum

Despite the availability of resources such as the AAIM Sub-internship Curriculum 2.0, 11 respondents (15.5%) noted their school does not have a structured AI curriculum. Forty-one percent (*n* = 29) of schools reported using at least one portion of the AAIM Sub-Internship Curriculum 2.0, while 47.1% (*n* = 33) have not used it at all. Fifty-eight percent (*n* = 41) of respondents offer didactics within the AI rotation. Table 2 details the specific curriculum components and teaching methods utilized.

**Table 2:** AI Curriculum

Survey question prompt	Answer choices	% response (*n*)
**Which components of the AAIM Sub-internship curriculum 2.0 do you use?**	Communicating effectively within healthcare teams	36.6% (26/71)
	Patient evaluation skills (recognizing sick vs not sick)	28.2% (20/71)
	Time Management	25.4% (18/71)
	Knowing when to ask for help	22.5% (16/71)
	Medical student wellness	11.3% (8/71)
**Teaching methods used other than clinical teaching on rounds/at bedside**	Didactics/lecture	57.7% (41/71)
	Informal sessions (i.e. AI report)	56.3% (40/71)
	Flipped classroom/asynchronous learning resources	31.0% (22/71)
	Patient log/passport	25.4% (18/71)
	Simulation	18.3% (13/71)
	No structured curriculum	15.5% (11/71)

### Clinical Responsibilities

Regarding AI clinical responsibilities, schools reported variability in patient load, night call or night shift, and daily clinical activities on the AI rotation. The average number of patients carried by AI students daily was 3–4 (74.3%, *n* = 52) with a maximum number allowed of 5–6 patients (71.4%, *n* = 50). Overnight call is required in only 8.5% (6/71) of schools, and night float is required in 23.9% (17/71). Eight institutions (11.3%) offer an optional night shift or night call experience. Table 3 lists the AI responsibilities.

**Table 3:** Clinical Responsibilities of AI Students

Survey question prompt	Answer choices	% response (*n*)
**Do students have either a required or optional night shift or night call during the AI?**	Yes	34.3% (24/70)
	No	42.9% (30/70)
	Varies by clinical site	22.9% (16/70)
**Average number of patients carried by AI student**	1–2	5.7% (4/70)
	3–4	74.3% (52/70)
	5–6	20% (14/70)
**Maximum number of patients carried by AI student**	3–4	18.6% (13/70)
	5–6	71.4% (50/70)
	7–8	7.1% (5/70)
	9 +	2/9% (2/70)
**Clinical Activities Allowed**	Assume primary responsibility for patients (carry own patients)	68/71 (95.8%)
	Write progress notes in EMR	67/71 (94.4%)
	Receive/give patient handoffs	66/71 (93.0%)
	Call consults	65/71 (91.5%)
	Actively participate in discharge planning with interdisciplinary staff	65/71 (91.5%)
	Answer nursing calls/pages/secure chats about own patients	64/71 (90.1%)
	Write admissions H&Ps in EMR	63/71 (88.7%)
	Assume responsibility for speaking to family members	62/71 (87.3%)
	Participate in end-of-life discussion/planning	62/71 (87.3%)
	Enter patient orders	61/71 (85.9%)
	Write discharge summaries in EMR	49/71 (69.0%)
	Perform verbal informed consents under direct supervision	47/71 (66.2%)
	Provide cross-coverage	31/71 (43.7%)

AI students are actively caring for patients on a daily basis, with 95.8% (68/71) of them carrying their own patients and 94.4% (67/71) writing daily progress notes in the EMR (see Table 3). Other important skills are also occurring with high frequency, such as giving/receiving handoffs (93%, 66/71), calling consults (91.5%, 65/71), actively participating in discharge planning (91.5%, 65/71), and answering nursing calls (90.1%, 64/71). Additionally, AI students are allowed to enter orders (85.9%, 61/71), participate in end-of-life discussions/planning (87.3%, 62/71), speak to family members (87.3%, 62/71), and write admission H&Ps in the EMR (88.7%, 63/71).

## DISCUSSION

The results of this national survey on the AI rotation during the fourth year of medical school provide insight into the current state of the AI as well as expectations and responsibilities of students on the rotation.

It is reassuring to see that all institutions surveyed require at least one AI rotation for graduation, and over a third of respondents require two or more. The requirement of at least one AI is an increase from prior CDIM survey results in 2001 (97.9%)^13^ and a statistically significant change from 2014 (90%, *p* < 0.01).^14^ It is especially encouraging given the importance of the IM AI within UME and in preparation for intern year.^22,23^ Only 10% (*n* = 7) of schools currently require students to complete an IM AI for graduation, which is a notable decrease from 2000 data when 26% of schools required an IM AI and 2017 data when 13% of schools required the IM AI.^13,15^ Despite this lack of a global requirement to complete an IM AI, many respondents report that students still complete an IM AI regardless of specialty, with 64.3% of schools noting that > 40% of their students complete an IM AI, which is unchanged from the 66.3% of respondents on the 2017 CDIM survey.^15^ This suggests that students (and their schools) recognize the importance of the rotation for their personal clinical development. While all schools require an AI rotation for graduation, 23% of schools allow an away AI to fulfill this AI requirement; this could present challenges in ensuring comparability as 15.5% of respondent institutions in this survey report not having a structured AI curriculum.

The typical length of the AI remains 4 weeks (98.6%) in duration. The number of AI specialties that fulfill the IM AI requirement (see Fig. 1) has grown over the years as compared to the most recent CDIM survey (in which the options specified included night float, general medicine wards, ambulatory medicine, and ICU).^15^ Providing additional subspecialties for the AI may provide a more comprehensive view of the scope of IM and also potentially increase AI capacity, allowing more students to complete an AI before their residency application is submitted. Ensuring clinical experiences and expectations are similar between services is essential to successfully broaden the offering of AI options.

Unsurprisingly, nearly all schools place their AI students on inpatient teaching services with residents and interns (94.4%). A significant number of schools also have an option for the student to work directly on a hospitalist-only service (42.3%), something which has not been asked on prior surveys and may allow more autonomy and 1-on-1 time with the attending physician than traditional ward teams. Rarely is an exclusively outpatient rotation counted as an AI, but 8.5% of schools do allow this option (see Fig. 1 for a breakdown of clinical experiences).

A total of 57.8% of respondents offer didactics within the AI rotation, which is a noticeable increase (*p* < 0.001) from 30.9% in 2001.^13^ Of these, 56.3% include informal AI sessions (such as AI report). The AAIM Sub-internship Curriculum 2.0 was published in 2018,^9,24^ yet 47.1% of schools do not utilize any part of it and 11.4% are unsure whether they use it. This is similar to the 2005 CDIM annual survey, in which 35% of schools used the original CDIM Sub-internship curriculum.^25^ Table 2 highlights schools’ use of the curriculum components and teaching methods utilized. The most utilized components are communicating effectively within healthcare teams (36.6%) and patient evaluation skills: recognizing sick vs. not sick (28.2%). Since program directors noted organization, time management, and prioritization skills as essential skills desired of new interns,^26^ these are areas that need improvement nationally to allow students to carry more patients daily under appropriate supervision. This is also a prime opportunity to incorporate time management tips, as suggested by the AAIM Sub-internship Curriculum 2.0,^9,24^ and promote student self-reflection to improve clinical efficiency in preparation for intern year and the potentially larger patient load. Despite this available resource, only 25.4% of respondents use the Sub-internship Curriculum 2.0 for teaching time management. Notably, 15.5% (*n* = 11) of schools still do not report having a structured AI curriculum, though this is an improvement from previously cited figures from surgical AI rotations data from 2013 and an improvement from nearly two-thirds of IM programs without a curriculum in 2005.^11,25^

Prior recommendations suggest that IM AIs should carry a minimum of 3–5 patients per day.^22^ This recommendation may be one explanation why 74.3% of respondents noted the *average* number of patients carried per day by AI students was 3–4 patients, while 20% of respondents stated students carried an average of 5–6 patients per day. In preparation for intern year, students on the AI rotation should strive to carry as many patients as possible to gain efficiency and learn time management skills. The majority of our survey respondents noted the *maximum* was 5–6 patients per day (71.4%). Compared to unpublished data from the 2012 CDIM survey, the maximum number of patients carried by AI students in our survey has decreased, with 20.8% of 2012 respondents reporting the maximum as 7–8, whereas in our survey only 7.1% reported the same maximum (*p* < 0.01).^27^ While ACGME guidelines state that an intern could be responsible for carrying up to ten patients per day^28^, recent studies suggest that the average intern patient census could be as low as 5–6 patients per day.^29,30^ Despite the potential for a lower daily patient census for interns, our study still demonstrates a gap in that most respondents report their AI students’ average daily census was 3–4 patients. Ultimately, it should be a goal to give AI students the opportunity to carry more patients, work on efficiency, and become more organized to be as prepared as possible by graduation.

Performing and receiving patient handoffs increased significantly from prior CDIM surveys to 93.0% of respondents compared to 45.9% in 2001 (*p* < 0.001).^13^ This increase is encouraging given handoffs is a clinical activity interns will perform on the first day of intern year. The increase in students performing patient handoffs may be a result of the incorporation of the Core EPAs into AI curricula and changing institutional cultures.^31,32^ Conversely, only 43.7% of respondents noted that AI students provided cross-coverage (EPA 10), which was similar to the 2001 data (51%) and 2012 CDIM survey (54%) (*p* < 0.05).^13,27^ Limited opportunities for cross-coverage risks leading to insufficient skill development in patient care.

In addition to the survey results showing the majority of IM AI students are not allowed to provide cross-coverage, only 33.8% of respondents require or offer an optional night shift or night call on the rotation. This is a significant gap in experience that students would benefit from in preparation for intern year, owing to the unique opportunities provided by an overnight call. Our data is similar to the 2014 CDIM survey results showing around 40% offered any overnight experience.^33^ The 2017 CDIM survey revealed 12–14% of respondents required overnight call and/or night float while 48% did not require it.^15^ The number of schools that do not offer a night float or call experience remained unchanged from 2017 data at 42.9% vs. 47.7% respectively. This is in stark contrast to 77% of students formally integrated into “call schedules” in 2001.^13^ The lack of opportunities for skill development in high-stress clinical situations such as night float or night call and cross-coverage could have implications on patient outcomes when these students are interns and therefore warrants significant and urgent attention.

### Limitations

While the institutional class size breakdown of our survey respondents mirrored the most recent CDIM national survey suggesting our survey was representative of the overall CDIM community of schools, our response rate of 51% may limit generalizability of the results. Given similarity of responses to identical questions between the surveys, we infer our survey results are a fair representation of the CDIM community of schools. Since variability in the wording and addition of new questions compared to prior CDIM surveys, the ability to compare our results to prior surveys was somewhat limited. Our survey focused on IM AI rotations; therefore, our results may differ from AI rotation trends in other specialties. Our study participants did not include osteopathic respondents, which appears to be an effect of how the study was distributed via the AAIM listserv. Since osteopathic schools graduate many students each year, it would be important to also gather data on the AI in those schools on further surveys.

### Future Directions

This manuscript focused on structural and curricular components of the AI rotation. We plan to analyze Core EPAs and other methods of assessment from our survey in future manuscripts. Our results can help inform AI directors of the current trends in AI curricula and expectations, serving as a needs assessment within individual institutions as well as call for reform nationally.

## CONCLUSION

This study represents a comprehensive and updated review of the IM AI since 2017. Most medical students still value the IM AI regardless of their planned residency and elect to take this AI even if not required. Unfortunately, students manage a relatively small number of patients daily but are frequently given many intern-level responsibilities such as writing notes, placing orders, answering nursing calls, performing handoffs, calling consults, and participating in discharge planning. Our data confirms the ongoing trend of fewer institutions requiring or offering night float components to their AI rotation, at the expense of less opportunities for students to obtain cross-coverage experience. Despite the freely available AAIM Sub-internship Curriculum 2.0 that addresses key skill areas program directors seek from their incoming interns, it remains underutilized nationally. While our data demonstrate several positive trends towards allowing students to undertake more clinical responsibilities on their AI rotation, there remains room for improvement in standardizing the AI experience and expectations to better prepare our medical students for intern year and better facilitate the critical UME to GME transition.

## Supplementary Information

Below is the link to the electronic supplementary material.

Supplementary file1 (PDF 76 KB)
