# Supplementary material for: Internal Medicine Acting Internship Trends in Rotation Structure and Student Responsibilities: Results from a 2023 National Survey
Source: J Gen Intern Med. 2024 Jul 29;40(1):287–93. doi: 10.1007/s11606-024-08897-2 (PMC11780068; doi:10.1007/s11606-024-08897-2)
Supplement: Supplementary file 1 — Supplementary file1 (PDF 76 KB) [file 11606_2024_8897_MOESM1_ESM.pdf]

Examining the current state of the internal medicine acting internship (or sub-internship) in undergraduate medical education

We are conducting a study to examine the current state of the internal medicine acting internship (AI) rotation (also known as the sub-internship) at the 155 LCME-accredited medical schools. By gaining a better understanding of these AI rotations and learning about their similarities and differences, we hope to be able to make recommendations about the structure, components, and grading in this rotation. This survey is NOT associated with the CDIM Annual Survey of Core Medicine Clerkship Directors and is NOT being fielded by the Alliance: it specifically focuses on the acting internship. Therefore, the questions are different and the survey is shorter. If you are not an acting internship director, we ask that you forward the survey participation URL to the appropriate person(s) at your institution. Please note that this study has been deemed IRB-exempt by the Florida Atlantic University Institutional Review Board. Participation in the survey is voluntary, and completing the survey implies consent to participate.

Name of institution (for tracking completion only)

____

What is the size of your 2023 graduating class?

- ◯ 1-50
- ◯ 51-80
- ◯ 81-120
- ◯ 121-200
- ◯ 201-220
- ◯ 221-250
- ◯ >250

How many AI rotations does your institution require for graduation?

- ◯ 0
- ◯ 1
- ◯ 2
- ◯ 3 or more

If you require an AI, can an away rotation fulfill the AI requirement?

- ◯ Yes
- ◯ No
- ◯ Unsure

How many weeks long is an individual AI rotation?

____

If your institution has an AI rotation graduation requirement, does your institution require an AI in internal medicine for all students regardless of specialty?

- ◯ Yes
- ◯ No
- ◯ Unsure
- ◯ N/A (we do not require an AI for graduation)

Which AI specialties are available within internal medicine? (Select all that apply)

- ☐ IM wards/General medicine
- ☐ Heme/Onc
- ☐ GI/Hepatology
- ☐ Cardiology
- ☐ Pulmonary
- ☐ ICU
- ☐ Nephrology
- ☐ Infectious Diseases
- ☐ Med-Psych
- ☐ Med-Peds
- ☐ Palliative
- ☐ Other
- ☐ Outpatient

What is the setting of the IM AI rotation at your institution?	◯ Entirely Inpatient<br>◯ Entirely Outpatient<br>◯ Equal mix of inpatient and outpatient<br>◯ Primarily inpatient with some outpatient<br>◯ Primary outpatient with some inpatient
What is the structure of your IM AI rotation? (select all that apply)	☐ Integrated onto teaching service with resident/interns<br>☐ Hospitalist only<br>☐ Outpatient faculty only<br>☐ Outpatient teaching clinic with residents/interns
Approximately what percentage of 4th year students complete an AI in internal medicine, regardless of requirement status?	◯ 0<br>◯ 1-20%<br>◯ 21-40%<br>◯ 41-60%<br>◯ 61-80%<br>◯ 81-100%
Do your AIs rotate at satellite campuses?	◯ Yes<br>◯ No
Have you utilized any portion(s) of the AAIM Subinternship curriculum 2.0 to incorporate new curriculum into the internal medicine acting internship?	◯ Yes<br>◯ No<br>◯ Unsure
Which of the AAIM subinternship curriculum 2.0 concepts have you adapted as part of your internal medicine AI? (select all that apply)	☐ Time management<br>☐ Communicating effectively within healthcare teams<br>☐ Patient evaluation skills: Recognizing sick vs non-sick<br>☐ Knowing when to ask for help<br>☐ Medical student wellness
Aside from clinical teaching on rounds and at bedside, what teaching methodologies does the AI at your institution employ as part of your curriculum? (select all that apply)	☐ didactics/lecture<br>☐ flipped classroom/asynchronous learning resources<br>☐ formative objective structured clinical evaluations (OSCEs)<br>☐ informal sessions (i.e. AI morning report)<br>☐ simulation<br>☐ patient log/passport<br>☐ no structured curriculum<br>☐ other
Please describe what other teaching methodologies your institution employs in the AI.	__________________________________

Which of the following clinical activities are students allowed/required to complete (select all that apply):	☐ answer nursing calls/pages/secure chats about own patients<br>☐ call consults<br>☐ perform verbal informed consents under direct supervision<br>☐ assume primary responsibility for patients (carry own patients)<br>☐ receive/give patient handoffs<br>☐ provide cross-coverage<br>☐ enter patient orders<br>☐ write admission H&Ps in EMR<br>☐ write progress notes in EMR<br>☐ write discharge summaries in EMR<br>☐ actively participate in discharge planning with interdisciplinary staff<br>☐ assume responsibility for speaking to family members<br>☐ participate in end-of-life discussion/planning
Do students have either a required or optional night shift or night call during the AI?	◯ Yes<br>◯ No<br>◯ Varies by clinical site
On the AI rotation, which of the following describes night call? (select all options that apply)	☐ Overnight call required (i.e. 24hr call)<br>☐ Night float required (i.e. night shift)<br>☐ Night experience is optional<br>☐ Varies by clinical site
What is the AVERAGE number of patients carried per day by an AI student?	◯ 1-2<br>◯ 3-4<br>◯ 5-6<br>◯ 7-8<br>◯ 9 or more
What is the MAXIMUM number of patients carried per day by an AI student?	◯ 1-2<br>◯ 3-4<br>◯ 5-6<br>◯ 7-8<br>◯ 9 or more
Do the evaluators at your institution receive training on bias?	◯ Yes<br>◯ No<br>◯ Unsure
How are grades assigned/determined for the AI rotation?	◯ individual (i.e. course director)<br>◯ grading committee
Which of the following grading structures is currently being utilized in the IM AI at your institution?	◯ Pass/Fail (i.e. competency achieved vs not achieved)<br>◯ Tiered-grading scale (i.e. Honors, high pass, pass OR A, B, C, etc)<br>◯ Hybrid pass/fail and tiered (i.e. Honors, pass, fail OR Pass with distinction, Pass, Fail)
Please give your grade distribution (%) for the past academic year on the acting internship	______________________________

What are the primary assessment methods being utilized in the internal medicine AI that factor into the overall grade designation? (select all that apply)

- ☐ Workplace-based assessments (direct observations)
- ☐ Standardized written exam (i.e. NBME advanced clerkship exam)
- ☐ Institution-specific written exam (homegrown)
- ☐ Oral exam
- ☐ Narrative comments on evaluation
- ☐ Criterion/competency-based rubric
- ☐ Objective Structured Clinical Evaluation (OSCE)
- ☐ 360 evaluations (such as nurse, SW, or fellow student evaluations)
- ☐ Grading of submitted write-ups (H&Ps, notes, etc)
- ☐ Simulation activities

Assessment/Evaluation Methods:

Please select all that apply from below. Please note, some options may be both formative and summative. Some options may be requirements while others may be optional.

	Formative	Summative	Requirement	Optional	N/A
Workplace-based assessments (direct observations)	☐	☐	☐	☐	☐
Standardized written exam (i.e. NBME advanced clerkship exam)	☐	☐	☐	☐	☐
Institution-specific written exam (homegrown)	☐	☐	☐	☐	☐
Oral exam	☐	☐	☐	☐	☐
Summative narrative comments	☐	☐	☐	☐	☐
Criterion/competency-based rubric	☐	☐	☐	☐	☐
Objective Structured Clinical Evaluation (OSCE)	☐	☐	☐	☐	☐
360 evaluations (such as nurse, SW, or fellow student evaluations)	☐	☐	☐	☐	☐
Grading of submitted write-ups (H&Ps, notes, etc)	☐	☐	☐	☐	☐
Simulation activity(s)	☐	☐	☐	☐	☐

Do you feel that grade inflation in the acting (sub) internship is present at your institution?

- ○ Yes
- ○ No
- ○ Unsure

Please select reasons you feel grade inflation occurs in your institution.

- ☐ People are uncomfortable giving negative feedback
- ☐ Don't want to discourage student from medicine
- ☐ Students doing an AI in medicine are inherently interested therefore deserve high grades
- ☐ Do not want to penalize student if other students are getting high grades
- ☐ Students expect a high grade in this rotation
- ☐ Don't want student to complain or contest their grade
- ☐ Felt guilty for not spending more time with student, more time teaching
- ☐ Fear of repercussions (legal)
- ☐ Faculty form good relationships with students and therefore rate them highly
- ☐ Evaluation data is not discerning enough to know true performance
- ☐ Other

Please list the "other reasons" for grade inflation

____

What effect would providing more transparency of student assessments and competencies achieved from the AI rotation(s) for inclusion on the MSPE have on each student's chances of successfully matching into an internal medicine residency?

- ○ No effect
- ○ Harmful effect (less chance of matching)
- ○ Positive effect (higher chance of matching)
- ○ Unsure

What effect would providing more transparency of student assessments and competencies achieved from the AI rotation(s) for inclusion on the SEL/Chair's letter have on each student's chances of successfully matching into an internal medicine residency?

- ○ No effect
- ○ Harmful effect (less chance of matching)
- ○ Positive effect (higher chance of matching)
- ○ Unsure

Does your AI rotation currently incorporate Core EPAs into the teaching and/or assessment?

- ○ Yes
- ○ No
- ○ Unsure

What Core EPAs are taught as part of your AI curriculum? (select all that apply)

- ☐ Gather a history and perform a physical examination
- ☐ Prioritize a differential diagnosis following a clinical encounter
- ☐ Recommend and interpret common diagnostic and screening tests
- ☐ Enter and discuss orders and prescriptions
- ☐ Document a clinical encounter in the patient record
- ☐ Provide and oral presentation of a clinical encounter
- ☐ Form clinical questions and retrieve evidence to advance patient care
- ☐ Give or receive a patient handover to transition care responsibility
- ☐ Collaborate as a member of an interprofessional team
- ☐ Recognize a patient requiring urgent or emergent care and initiate evaluation and management
- ☐ Obtain informed consent for tests and/or procedures
- ☐ Perform general procedures of a physician
- ☐ Identify system failures and contribute to a culture of safety and improvement

Which Core EPAs are assessed during the AI by means of direct observations (and associated workplace-based assessments)?<br>(select all that apply)	☐ Gather a history and perform a physical examination<br>☐ Prioritize a differential diagnosis following a clinical encounter<br>☐ Recommend and interpret common diagnostic and screening tests<br>☐ Enter and discuss orders and prescriptions<br>☐ Document a clinical encounter in the patient record<br>☐ Provide and oral presentation of a clinical encounter<br>☐ Form clinical questions and retrieve evidence to advance patient care<br>☐ Give or receive a patient handover to transition care responsibility<br>☐ Collaborate as a member of an interprofessional team<br>☐ Recognize a patient requiring urgent or emergent care and initiate evaluation and management<br>☐ Obtain informed consent for tests and/or procedures<br>☐ Perform general procedures of a physician<br>☐ Identify system failures and contribute to a culture of safety and improvement
Does your institution make entrustment decisions on any of the Core EPAs?	◯ Yes<br>◯ No<br>◯ Unsure
Which of the 13 core EPAs are given an entrustment decision? (select all that apply)	☐ Gather a history and perform a physical examination<br>☐ Prioritize a differential diagnosis following a clinical encounter<br>☐ Recommend and interpret common diagnostic and screening tests<br>☐ Enter and discuss orders and prescriptions<br>☐ Document a clinical encounter in the patient record<br>☐ Provide and oral presentation of a clinical encounter<br>☐ Form clinical questions and retrieve evidence to advance patient care<br>☐ Give or receive a patient handover to transition care responsibility<br>☐ Collaborate as a member of an interprofessional team<br>☐ Recognize a patient requiring urgent or emergent care and initiate evaluation and management<br>☐ Obtain informed consent for tests and/or procedures<br>☐ Perform general procedures of a physician<br>☐ Identify system failures and contribute to a culture of safety and improvement
Do AI notes count as billable documents at your institution with appropriate attestation?	◯ Yes<br>◯ No<br>◯ Unsure<br>◯ Varies by clinical site
Does your MSPE include any information regarding 4th year rotations?	◯ Yes<br>◯ No<br>◯ I don't know
Does your institution's MSPE provide the grade distribution for the medicine AI rotation?	◯ Yes<br>◯ No<br>◯ I don't know

Does your institution's SEL/Chair's Letter include the grade distribution for the medicine AI rotation?

◯ Yes
◯ No
◯ I don't know
